# Investigation of the subcellular architecture of L7 neurons of *Aplysia californica* using magnetic resonance microscopy (MRM) at 7.8 microns

**DOI:** 10.1038/srep11147

**Published:** 2015-06-10

**Authors:** Choong H. Lee, Jeremy J. Flint, Brian Hansen, Stephen J. Blackband

**Affiliations:** 1 Department of Neuroscience, University of Florida College of Medicine, Gainesville, FL; 32611; 2 Mcknight Brain Institute, University of Florida, Gainesville, FL; 32611; 3 Center of Functionally Integrative Neuroscience (CFIN), Aarhus University, 8000 Aarhus C, Denmark; 4 National High Magnetic Field Laboratory, Tallahassee, FL; 32310

## Abstract

Magnetic resonance microscopy (MRM) is a non-invasive diagnostic tool which is well-suited to directly resolve cellular structures in *ex vivo* and *in vitro* tissues without use of exogenous contrast agents. Recent advances in its capability to visualize mammalian cellular structure in intact tissues have reinvigorated analytical interest in aquatic cell models whose previous findings warrant up-to-date validation of subcellular components. Even if the sensitivity of MRM is less than other microscopic technologies, its strength lies in that it relies on the same image contrast mechanisms as clinical MRI which make it a unique tool for improving our ability to interpret human diagnostic imaging through high resolution studies of well-controlled biological model systems. Here, we investigate the subcellular MR signal characteristics of isolated cells of *Aplysia californica* at an in-plane resolution of 7.8 μm. In addition, direct correlation and positive identification of subcellular architecture in the cells is achieved through well-established histology. We hope this methodology will serve as the groundwork for studying pathophysiological changes through perturbation studies and allow for development of disease-specific cellular modeling tools. Such an approach promises to reveal the MR contrast changes underlying cellular mechanisms in various human diseases, for example in ischemic stroke.

Interest in MR signal behavior and characteristics in the cells of multicellular animals has gained momentum after the first demonstrated MR visualization of an animal cell: a frog egg in 1986^1^. Since then, advances in MRM hardware, MRM techniques and the use of higher magnetic fields have facilitated unprecedented differentiability of spatial information in biological and non-biological systems (reviewed in^2^)^3^,^4^ as well as spectroscopic analysis of intracellular regions in *Aplysia californica* L7 neurons^5^ and *Xenopus laevis* oocytes^6^. In the case of *Xenopus laevis*, cellular division of embryos has also been imaged using MRM techniques^7^,^8^. Previous MR microscopy studies have reported resolutions as high as 1 μm in-plane (with 75 μm thickness)^9^ and 3 μm isotropic^10^,^11^. Such refinement of MRI techniques has only recently permitted direct visualization of mammalian cells using this imaging modality. In diffusion weighted MRM scans of mammalian neurons^12^,^13^,^14^,^15^, one consistent finding is the hypointense signal of cell bodies and the initial segments of their processes. These findings suggest a high diffusivity in the perikarya and large neurites as compared to areas containing neuropil and the extracellular space. Such findings are contrary to what is often assumed in cellular modeling studies^16^,^17^. These MR microscopy techniques may improve our understanding of the mechanisms behind diffusion signal changes in ischemic stroke^18^, for example, by providing reference data for modeling and by enabling well controlled tissue perturbation studies with cellular level resolution in live perfused slices^19^. Direct measurement of MR diffusion signal characteristics in intracellular regions^20^ and their changes after chemically-driven energy failure were used as a cellular model for the diffusion signal increase in neural tissue observed during ischemic stroke^20^. Even with the insight offered by these studies, the origins of signal changes at the cellular level remain a subject of debate^21^. It should be noted, however, recent studies suggest plausible biological and physical mechanisms^22^,^23^ behind detected changes of diffusion signals that might be tested experimentally in the future with MRM.

Care must be taken to correctly identify—through correlative histology—specific tissues in the microscopic domain to ensure that the correct interpretation regarding cellular MR properties is made. Such determinations are based on meticulous sample preparation and precise placement in both imaging modalities—MRM and light microscopy—followed by rigorous post-imaging data comparison. Tissue-specific contrast and spatial information unique to each imaging method is generated using directly targeted methodologies. In the current study, we report MRM of *Aplysia* L7 neurons at resolutions as high as 7.8 × 7.8 × 15 μm^3^ which we believe to be the highest taken of this model to date. In addition, these MR data are presented in conjunction with correlative histology of the same samples detailing the exact spatial positions of the nuclear and plasma membranes. The MR resolution required to distinguish these boundaries is achieved using dedicated hardware: micro surface-coils^24^ to increase the signal-to-noise ratio and strong/fast switching imaging gradient coils (4.8 G/cm/A) to increase resolution and minimize diffusion times.

To the best of our knowledge, this is the first study using direct correlative histology to confirm the identity of intracellular structures in the neuronal soma of the sea slug *Aplysia californica*. Through identification of subcellular elements such as nucleus, cytoplasm, and cell membrane, with multi-modality examination, we have ensured the correct identification of subcellular structures in MR images through correlative histology. This ability is nontrivial as successful future modeling efforts in MR will depend heavily on our ability to correctly identify subcellular tissue elements. We hope the presented data and methodologies will contribute to the development of ever more accurate cellular models^25^,^26^,^27^ which themselves will be instrumental to correlating changes in MR signal characteristics with disease-specific pathology encountered in the clinic.

## Methods

### Cell isolation and sample preparation

Adult, marine gastropods, *Aplysia californica*, were obtained from the *Aplysia* Resources Center (University of Miami, FL) and L7 neurons along with additional satellite cells were isolated from the abdominal ganglia by gross dissection. This dissection proceeded without use of a collagenase/trypsin digestive solution to avoid any chemical interference or potential loss of satellite cells. Hemolymph from the sea slugs was extracted from the abdomen through the foot by a 21-gauge syringe needle. This fluid was then used as a surgical media and imaging buffer. Multiple ganglionic cells in the 40–70 μm diameter range were left intact and attached to the L7 neurons in order to assist in sample positioning. The isolated cells were fixed for a minimum of 24 hours in 4% formaldehyde. After 3X serial washes in PBS, a subset of neurons (usually 4–5) were selected and immersed in low-melting point agarose (22–110–617, Fisher) for high throughput sectioning. Next, neurons were sectioned using a vibratome (Ted Pella, Lancer series 1000) into slices of various thicknesses (25 to 100 μm). Delicate sample handling was practiced before and after MR scanning to ensure preservation of the sample slice through the selection processes (light microscopy), imaging (MRM), and subsequent histological validation (light microscopy). Selection of appropriate samples—i.e. those with intact, visible nuclear compartments following slicing—was conducted using a light microscope (Zeiss, Axioplan 2) prior to running MR microscopy to ensure these structures of interest were represented in the final MRM scans.

### Sample positioning and magnetic resonance microscopy (MRM)

**Figure 1:**
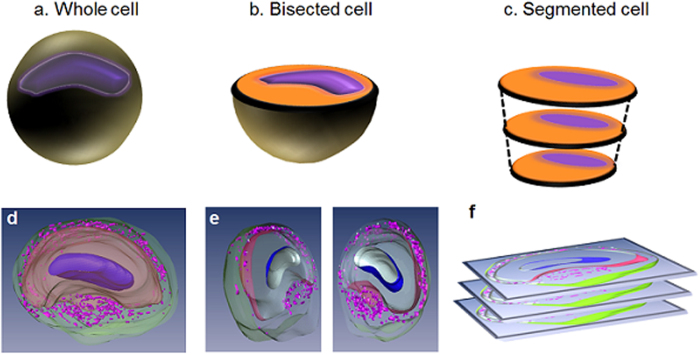
Schematic explaining our various sample preparation protocols. (**a**) The whole cell. (**b**) The bisected cell to expose the intracellular organelles. (**c**) The segmented model for facilitating histological correlation after MR imaging. The intracellular compartments are assigned as such: nucleus (blue dots), cytoplasm (yellow areas), and dense fibers (green) around the satellite cells. 3D reconstructed image of single neurons in each sample preparation respectively d-f. The 3D visualization was generated after interpolating multiple slices of 3D MRM images in Fig. 3a. Nucleus in the cell (dark purple), cytoplasm (beige), and satellite cells (purple) in the satellite cell region (green).

All MRM was carried out using a 600 MHz Oxford spectrometer equipped with a Bruker Biospin console and micro-5 imaging gradient providing strengths up to 3000 mT/m. We employed two different slicing methodologies as a way to image L7 neurons. The first included placing the cut surface of a bisected, fixed L7 neuron (Fig. 1b) directly in contact with the face of our 500 μm diameter micro surface-coil (Z76414, Bruker Biospin) thus retaining cellular organelles while still gaining imaging access to the neuron’s interior. The second method involved imaging pre-sliced sections (≥25 μm) of fixed neurons which was required to achieve positive, one-to-one image correlation with light microscopy.

**Figure 2:**
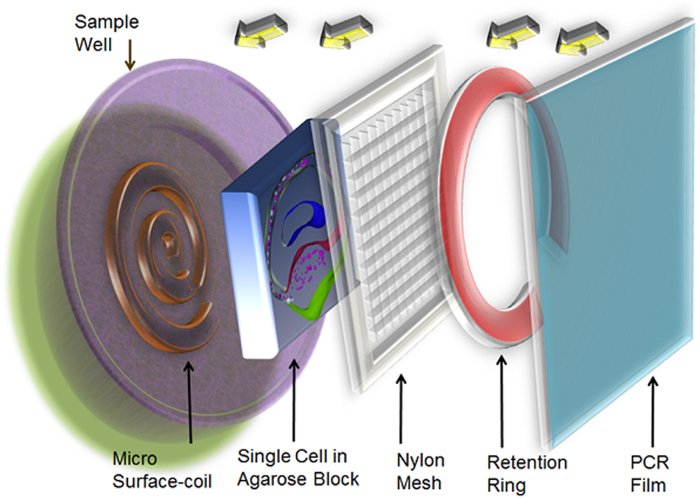
Schematic of sample placement in the radio-frequency (RF) coil well. The fixed cell slice embedded in the agarose block lies directly on top of the RF coil. The sample is then retained using nylon mesh and C-shaped retention ring. Finally the tissue well is tightly sealed by PCR film.

After positive identification of the L7 neuron was made using a dissecting scope (Zeiss, OPMI 1-FC), these neurons and surrounding cells were extracted by gross dissection. Following this excision, cell clusters were examined to ensure that the L7 cell’s plasma membrane was intact. The selected, intact samples underwent fixation in 4% formaldehyde solution overnight. To reduce the concentration of fixative prior to imaging, samples were washed in PBS through no less than four buffer changes. Bisection was achieved using a handheld scalpel and employed the largest of our cell samples to help reduce the technical difficulty. In an attempt to reveal subcellular structures, ultra-high resolution 3D images of these bisected cells were obtained. For the experiments conducted on bisected neurons (Fig. 1b), a sample holder was developed in-house to keep the cells from experiencing excess mechanical stress from water surface tension during placement of the tissue and PCR film (Fig. 2). Additionally, to aid in placement of the bisected cell into the comparatively large sample well, the diameter and depth of which are 5 mm and 500 μm, respectively, additional neighboring cells were left intact and attached to the neuron.

### 3D segmentation and histological comparisons

Three-dimensional, spin-echo datasets containing the nucleus, cytoplasm, and the tissue layer surrounding the L7 were reconstructed using image analysis software (Amira 5.4.0; Visage Imaging) so that subcellular structures could be displayed and appreciated from multiple viewpoints. For validating the identities of cellular compartments delineated using MR contrast, fixed L7 neurons suspended in 2 ~ 3% agarose solution were segmented as described in Fig. 1c and f. Using Nissl (cresyl violet) stain, we identified the location and boundaries of the intracellular cytoplasm in relation to other cellular compartments. After tissue-specific labeling of the cytoplasm in histological sections, these light microscopy images were compared to their MRM counterpart so that subcellular tissues could be correlated between images. In turn, DAPI stain (Vectashield, VectorLab H-1200) was employed to label the nuclear membranes of L7, neighboring cells, and various other satellite cells in close proximity to these. Finally, to define the boundary between the inside and exterior of the L7 neurons, the delineation of the cells’ plasma membranes was carried out using PKH 67 Fluorescent Cell Liner Kits dye obtained from Sigma Biosciences (MINI67-1KT). For the comparative analysis of proteolytic loosening effects, a surgical protocol requiring enzymatic digestion, i.e. 30 min to 1 hour immersion in the digestion medium, collagenase 3.5 mg/ml artificial sea water (ASW; NaCl: 460 mM; KCl: 10.4 mM; MgCl_2_: 55 mM; Hepes: 15 mM; pH = 7.8) and trypsin (33% 10X), was employed in order to loosen the ganglia sheath.

## Results

### Delineation of subcellular architecture in an *Aplysia californica* neuron using MR contrast

**Figure 3:**
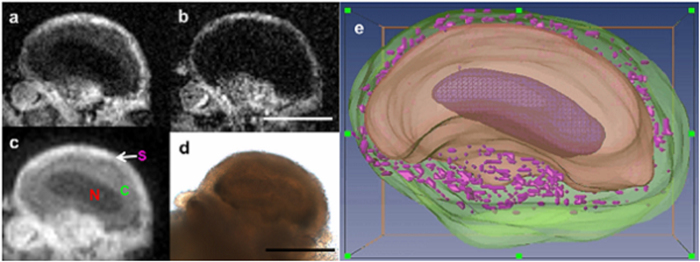
Direct correlation of *Aplysia* neuron morphology in MRM and histological images. (**a**) One slice from a 3D spin echo image of the entire fixed L7 neuron illustrating cellular compartments using a micro surface-coil at 600 MHz NMR spectrometer, MRM scan parameters: 3D spin echo sequence with TE/TR = 8.4/1000 ms, resolution = 7.8 × 7.8 × 15 μm^3^, FOV = 1 mm × 1 mm × 1 mm, matrix = 128 × 128 × 64, NEX = 10, acquisition time = 22 hrs 51 min. (**b**) The same slice at an increased TE of 12 ms. (**c**) Lower resolution image presenting the possible plasma membrane. MRM scan parameters: 3D spin echo sequence with TE/TR = 8.4/1000 ms, resolution = 12 × 12 × 15 μm^3^, FOV = 1.5 mm × 1.5 mm × 1 mm, matrix = 128 × 128 × 64, NEX = 6, acquisition time = 13 hrs. (**d**) Corresponding light microscopic image illustrating signal heterogeneity in the outer periphery and connective tissue. Nucleus (N), Cytoplasm (C), Satellite cell region (S), (**e**) 3D visualization generated by interpolating multiple slices of 3D MRM images. Nucleus (dark purple), cytoplasm (beige), and satellite cells (purple) in the satellite cell region (green). Scale bar = 300 μm.

**Figure 4:**
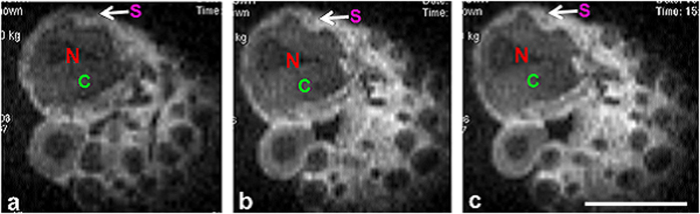
Three representative serial slices from a 3D spin echo MRM of the bisected L7 cell and neighboring, intact smaller cells. The innermost dark region corresponds to the nucleus (N) and the slightly brighter region immediately adjacent to it, the cytoplasm (C). The satellite cell region (S) exhibits the most intense signal. MRM scan parameters: 3D spin echo sequence with TE/TR = 8/1800 ms, resolution = 11.7 × 11.7 × 11.5 μm^3^, FOV = 1.5 × 1.5 × 0.74 mm, matrix = 128 × 128 × 64, NEX = 6, acquisition time = 24 hrs. Scale bar = 300 μm.

Figure 3 shows representative images of intracellular structures of the L7 neuron using endogenous MR contrast. In these T2-weighted images with an in-plane resolution of 7.8 μm (TE = 8 ms), intracellular structures were distinguishable due to their appreciably different signal-to-noise ratios (SNR): nucleus (SNR = 2.6 ± 0.6), cytoplasm (SNR = 5.7 ± 0.9), satellite cell region (SNR = 18.3 ± 1.9) (Fig. 3a). Upon increasing the TE to 12 ms, the nucleus could no longer be differentiated from the cytoplasm (Fig. 3b). Lowering the in-plane resolution to 12 μm (Fig. 3c) as a means of increasing signal improved the demarcation of the intracellular compartments in the same cell. Individual cells in the satellite cell region can be seen in the light microscopy image (Fig. 3d). Tissues were segmented and visualized using AMIRA 5.4.0 (Visage Imaging). The solid purple, innermost tissue corresponds to the nucleus while the cytoplasm is represented in beige (Fig. 3e). The satellite cell nuclei (small purple dots) are embedded in neuropil (transparent green). 3D spin echo images show that intracellular structures remained intact following bisection (Fig. 4). Contrast characteristics appear similar to those seen in Fig. 3(c). These structures share morphological features—nucleus, cytoplasm, and satellite cell layer—in common despite the differences in sizes, locations, etc.

### Histological confirmation of the MR-delineated subcellular architecture of an *Aplysia californica* L7 neuron

**Figure 5:**
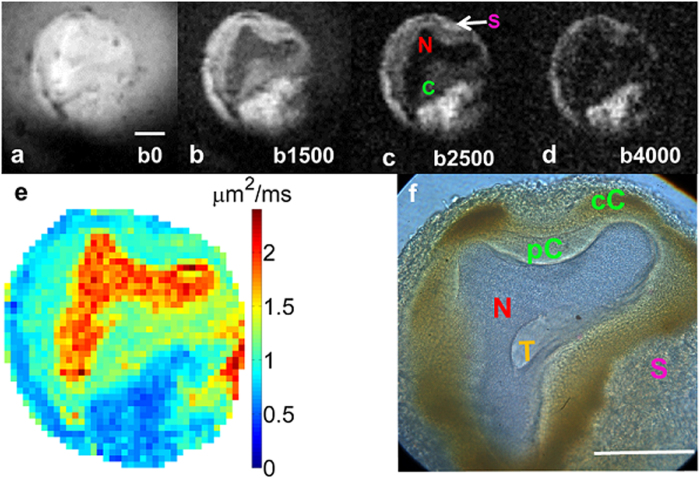
Diffusion weighted images of a slice of the single neuron embedded in agarose. MRM scan parameters: TE/TR = 20/2000 ms, resolution = 11.7 × 11.7 × 100 μm^3^, NEX = 28, acquisition Time = 2 hours, bandwidth = 50 kHz, read gradient amplitude = 784 mT/m, and phase gradient amplitude = 743 mT/m, b-values = (**a**) 0, (**b**) 1500, (**c**) 2500, (**d**) 4000 s/mm^2^ (**e**) ADC map in mm^2^/s, diffusion gradient separation (Δ)/ diffusion gradient duration (δ) = 11 ms/1 ms. (**f**) Light microscopy image of the same neuron showing five distinct subcellular tissue types at 40x magnification. Of particular note is the morphological delineation of more than two intracellular compartments consisting of nucleus (N), perinuclear cytoplasm (pC), cortical cytoplasm (cC) enriched with mitochondria and lipochondria functioning as photoreceptors, and possibly trophospongium (T), which is glial contact to the large neuron via membrane invagination. Scale bar is 100 μm.

**Figure 6:**
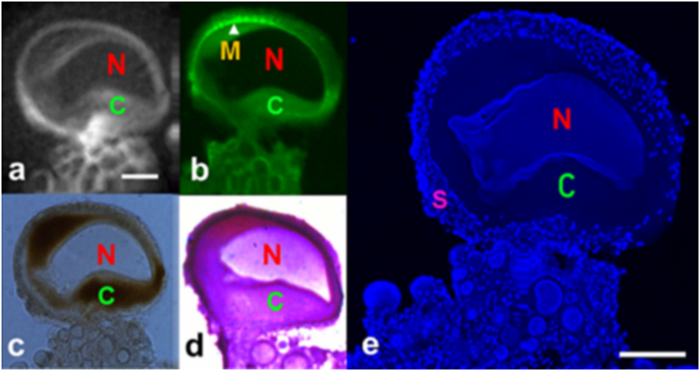
Direct correlation of cellular architecture of single neurons of Aplysia californica in MRM and histological images. (**a**) 2D diffusion-weighted images at the in-plane resolution of 7.8 μm^2^ with 50 μm thick slice at b = 1500 s/mm^2^. (**b**) Green fluorescent colored PKH 67-stained plasma membrane. (**c**) Bright-field microscopy. (**d**) Nissl-stained cytoplasmic region of the adjacent slide. (e) DAPI-stained nucleic acid in the nucleus in a single neuron; smaller cells and numerous tiny satellite cells around the periphery of the neuron. MR scan parameters: TE/TR = 20/2000 ms, resolution = 11.7 × 11.7 × 150 μm^3^, NEX = 100, FOV = 1.5 × 1.5 × 0.15 mm, acquisition time = 2 hours, b-values = 1500 s/mm^2^, diffusion gradient separation (Δ)/diffusion gradient duration (δ) = 5 ms/1 ms. Nucleus (N), cytoplasm (C), plasma membrane(M), and satellite cells(S). Scale bar is 100 μm.

Figure 5 displays water diffusion characteristics in the cellular substructures of a L7 neuron (75 μm thick section). In the image with the least amount of diffusion weighting (b = 191 s/mm^2^ in Fig. 5a), signal in the L7 neuron (SNR of 61) is similar to the PBS (SNR of 51) surrounding the cell. Diffusion weighting above 1000 s/mm^2^ (1500 s/mm^2^ in Fig. 5b and 2500 s/mm^2^ in Fig. 5c), results in enough contrast to delineate multiple intracellular structures such as the nucleus, cytoplasm, and outer rim of tissue containing satellite cells. At b-values higher than 4000 s/mm^2^, all diffusion signal from inside the L7 became hypointense while signal in the outer rim of satellite cells remained hyperintense (Fig. 5d). An ADC map calculated from a series of diffusion weighted images is shown in Fig. 5(e). The spatial location of these satellite cells is shown to be confined to the outermost tissue layer in our image which, as is detailed in the correlative histology, lies beyond—i.e. outside—the cell membrane of the L7 neuron (Fig. 5f). Light microscopy images of a single L7 neuron showed five distinguishable subcellular compartments at 40x magnification. Of particular note here is the detailed morphological delineation of more than two intracellular compartments: the nucleus (N), the peri-nuclear cytoplasm (pC) which contains mitochondria and rough endoplasmic reticulum^28^,^29^, the cortical cytoplasm (cC) enriched with lipochondria, lysosomes^30^,^31^ and enzymes controlling light activation in the photoreceptors^32^, and the satellite cell layer (SCL) in which satellite cells including glia were embedded. In addition, a centralized structure is visualized in our light microscopy which is most likely the trosphospongium (T). It is an invagination of the glial terminal formed to support the energy requirements for large neurons. This structure is unique to gastropod mollusks (slugs and snails) and hirudinean annelids (leeches)^33^,^34^,^35^,^36^. Cellular features were highly delineated in brightfield microscopy images relative to MRM, thus aiding in identification of structures observed in MR images. For delineation of the anatomical border separating the intracellular compartment of the L7 from the neuron’s exterior, PKH 67 fluorescent dye (Sigma Biosciences, MINI67-1KT) was used to stain the cell membrane (Fig. 6b). Green fluorescent signal highlights the plasma membrane which correlates spatially with the separation between the cytoplasmic compartment and satellite cell layer seen in the corresponding MRM (Fig. 6a). From the unstained light microscopy image in Fig. 6(c), one can appreciate the different opacities of the subcellular tissues and compartments: e.g. translucent nucleus and opaque cytoplasm and satellite cell layer. By employing Nissl stain (Fig. 6d), the location and boundaries of the cytoplasm was verified. This method highlighted the cell body, specifically the endoplasmic reticulum, in a blue or purple-blue hue. Nuclei-specific DAPI stain clearly identified the nuclear compartment in the single L7 neuron and satellite cells embedded in the surrounding tissue layer (Fig. 6e).

**Figure 7:**
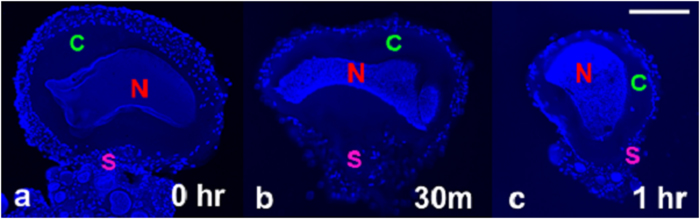
Temporal changes of three different single neurons following our enzymatic digestion protocol. Exposure time: (**a**) 0 min, (**b**) 30 min, (**c**) 1 hour. Of particular note is the morphological change occurring around the neuron such as the loss of plasma membrane integrity, thinner layers of satellite cells which are inhomogeneous, and increased staining intensity in the large nuclei of the treated sample (b and c). Nuclei of satellite cells distributed in the satellite cell region were also labeled by the DAPI stain. Scale bar is 100 μm.

The reduction of the satellite cell layer thickness and cell density following digestive enzyme treatment was revealed through DAPI staining (Fig. 7). Proteolytic enzyme degradation resulted in the digestion and subsequent loss of the layer containing satellite cells (Fig. 7b). From our examination of digested samples stained with DAPI at three different time points (0, 0.5, and 1 h), the effects of the enzymes trypsin and collagenase were shown to be most prominent in the satellite cell layer and increased in severity with time (Fig. 7b,c).

## Discussion

Because of the L7’s size (300–500 μm) and surgical accessibility of the *Aplysia’s* abdominal ganglion^37^, this cell model is well suited for MRM studies. Using micron-scale resolution, MR signal characteristics were examined in subcellular compartments of the *Aplysia californica* L7 neuron. To the best of our knowledge, this study reports the first instance in which the identities of specific subcellular tissues in the L7 neuron visualized by MRM are confirmed through correlative histology. Such data collection was made possible by fixation and careful sectioning of the L7 neuron as this allowed direct contact between the intracellular compartments with the micro surface-coil. After increasing the accessibility of the intracellular domain by using either a bisected or segmented sample preparation protocol, the cell’s contents were optimally positioned in the center region of the RF detector to provide the highest sensitivity. Such methodology was crucial for generating clearly delineated tissue types in our MRM thus making definitive histological confirmation of these structures possible.

The surgical protocol used in previous studies employed digestive enzymes (collagenase and trypsin) which helped loosen exterior tissues surrounding the L7 cell so as to reduce the chances of cell breakage during excision. As a result of such enzymatic treatment, satellite cell loss occurred around the L7. In previously conducted SEM studies^38^,researchers pointed out the potential for loss of satellite cells following proteolytic processing which we observed during our examination. However, in our revised protocol in this study, cell isolation was completed without collagenase and trypsin treatment for the purpose of preserving the structural integrity of the satellite cell layer. Furthermore, in order to reduce any potential loosening of this tissue layer leading to the loss of satellite cells^38^,^39^ and potential changes in the tissue’s diffusion properties—such as those reported in the mammalian system after relaxin treatment^40^—artificial media was avoided whenever possible by substituting the animals’ hemolymph^41^. An unintended benefit of using methodologies which effectively preserve multiple ganglionic neurons was that the intact basement membrane and surrounding cells served as an effective positioning tool allowing for careful placement inside the RF micro coil. Additionally, the use of a surface coil as opposed to solenoidal coils^3^,^42^,^43^,^44^,^45^ allowed for more effective manipulation of tissue orientation.

Remnants of satellite cells following proteolytic processing could potentially interfere in interpretation of lower-resolution MR resulting in difficulties with compartmental identification and subsequently leading to erroneous assignment of quantitative values: e.g., relaxation values and apparent diffusion coefficients (ADC). For example, in a previous study validating cellular diffusion anisotropy, Hsu *et al*. (1997) concluded that the apparent diffusion isotropy came from subcellular diffusion isotropy rather than averaging effects while Grant *et al*. (2001) demonstrated anisotropic diffusion only in the cytoplasm regions^44^,^45^. Both publications suggested the need for further investigation to elaborate their results by aid of higher sensitivity and specificity. Compartmental identification was not validated in earlier studies in part due to partial volume averaging effects resulting from large slice thicknesses, e.g. 15 × 15 × 100 μm^3^, 40 × 40 × 150 μm^3^ 44,45. Furthermore, such inherent limitations were present when monitoring changes in intracellular water diffusion following hypotonic perturbation by 20%^43^. Recently, using 25 μm isotropic resolution and a lethal 33% hypotonic shock, MR signal behaviors were examined in an isolated abdominal ganglia cell^46^. The observed threshold effects for cell swelling events—e.g. no change in soma ADC following a 20% hypotonic perturbation but increase in soma ADC by 32% following a 33% hypotonic perturbation—might result from overcoming innate structural and functional protection against external perturbation by osmotic regulation^28^,^38^,^47^. Ignoring factors such as natural sample variability and the degree of osmotic challenge of artificial seawater (ASW), the reported threshold effect might reflect the importance of other factors whose parameters have yet to be determined such as specificity and sensitivity.

Although fixation has been shown to change the MR relaxation properties of tissue^48^, employing fixed samples allowed us to access novel morphological information through high resolution studies. Our work demonstrates that previous studies disregarded or misidentified the satellite cell lamina^5^,^42^,^44^. Issues regarding tissue ambiguity are further exacerbated given morphological differences, e.g. spherical, oval, amorphous, or variability in position of nuclei, which differ according to cell function and maturation^28^. To elucidate the true boundaries of tissue lamina for positive tissue identification, histological correlation was essential. The methodologies employed in the current study were designed to allow for direct, unambiguous confirmation of the intracellular compartments in the L7 neuron.

T2 contrast inhomogeneity in the nucleus (in contrast to the homogeneity in the cytoplasm) reported previously^3^ can also be addressed in light of new findings. Subcellular structures are clearly demarcated using T2-weighted 3D spin echo imaging (Figs 3,4). Specifically, contrast between the nucleus and cytoplasm was present in the 8 ms TE images of Fig. 3a, but merged to match cytoplasmic signal contrast in images collected with a 12 ms TE (Fig. 3b). The T2 contrast mechanism demonstrated here was not intended for quantification, but for contrast-based delineation of the intracellular structures. Such contrast requires strong imaging gradients resulting in significant diffusion weighting effects^49^,^50^. Additional care must also be taken to interpret the MRM of fixed cells since chemical fixation is known to cause a reduction in T2: frog eggs^51^, mouse^52^, and human nervous tissue^48^,^53^,^54^.

In previous studies employing whole, intact cells in which neurons were imaged in solenoid coils, the tissue thickness and high through-plane sample heterogeneity prevented accurate light-based histological confirmation of the intracellular structures after MRM^3^,^42^,^43^,^44^,^45^. In the earliest MR study using the L7 cell model, Schoeniger *et al*. (1994) defined the region corresponding to the nucleus based only on MRM collected with criteria derived to attain images during the cells’ viability period^42^. This necessitated scans with limited resolution (20 × 20 × 100 μm^3^) that suffer from substantial partial volume effects. In addition, by employing a relatively long TE value (15.15 ms) as the minimum echo time, it is likely that excessive T2 weighting would result in the blending of the nuclear and cytoplasmic compartments, thus making their delineation difficult. Therefore, it was a natural extension of these early studies to derive methodology that would eliminate these limitations. The improved resolution and sensitivity generated by using higher magnetic fields, stronger gradients and micro surface coils coupled with improved accessibility to the interior of fixed L7 cells allowed us to achieve direct correlative results between our MRM and histological analysis. Such techniques are critical to future efforts focused on developing accurate tissue modes.

In a previous study, the satellite cell layer was visualized using scanning electron microscopy (SEM)^38^ and found to be diminished. The authors suggested two possible causes for the loss of satellite cells: dehydration and/or the dissection process. In Fig. 7, cells not subjected to enzymatic digestion contained a substantial satellite cell layer, but after 1-hour treatment the remaining satellite cells were non-uniformly distributed around the periphery of the L7 neuron as previously reported^38^,^55^,^56^. This effect is especially apparent along the contour of the cell membrane. In Fig. 5, diffusion signal intensity between intracellular regions changed from hyper to hypointense between b = 0 and b = 1500 s/mm^2^. This regional hypointensity comes from the relatively faster diffusion occurring in the nucleus and cytoplasm as compared to the satellite cell layer.

The origin of reduced ADC in the L7’s satellite cell layer is due to its characteristic cellular composition and architectural organization. Presumably, the high content of water and glycogen in satellite cells used as nutrient stores for the L7 could be restricted in the highly dense anastomotic mesh. Water in this region could become tightly bound within fibers whose sizes are approximately 0.5 ~ 2 μm as demonstrated previously in SEM images^38^. The mean displacement distance of 5 μm coupled with the effective diffusion time, 4 ms, was still too large to pinpoint the primary cause of restriction effects whether they be satellite cells, extracellular mesh, or a mixture of both. Still, there might be a physical correlation with the slow diffusing water pool described recently by Le Bihan as a bound water layer on the interior of the cell membrane^21^. It is of note that the gradient strengths employed in our study (784 mT/m in frequency encoding and 743 mT/m in phase encoding direction), exceeded the values necessary to avoid blurring problems caused by diffusion-limited resolution^2^,^4^,^57^. With the stronger and faster-switching gradients employed in the current study, the exact location and, more importantly, the boundaries of tissue layers responsible for water restriction inside and outside the L7 neuron were visualized.

The current work validates the identities of multiple microscopic tissue structures including the nucleus, cytoplasm and satellite cell layer of the L7 neuron. Correlative histology allowed for the delineation of the plasma membrane thus giving a physical boundary in both datasets which indicated that the satellite cell layer was outside the boundaries of the L7’s intracellular compartment: an insight not readily apparent upon viewing the MR microscopy image in isolation. Knowledge of the MR properties of cellular microstructure can be used to develop accurate models of disease-specific changes manifesting at the cellular level. Equipped with higher resolution and sensitivity, previous perturbation studies^43^ should be revisited so that data can be provided to improve modeling efforts. Such studies conducted on intact neurons with sufficient support from an intact satellite cell layer might help us to better understand the mechanisms and dynamics behind the decrease in tissue ADC observed in ischemic stroke^58^,^59^, as well as the interpretation of MR signals in healthy and pathological tissue.

### Conclusions and Future Work

By virtue of generating correlative histology from samples which underwent diffusion weighted MRM, this study was able to positively identify specific subcellular tissues in the L7 neuron of the gastropod *Aplysia californica*. Such validation is important to ensure accurate representation of tissue compartments of aquatic models as they are applied to mammalian models in terms of their similarity or dissimilarity in subcellular signal properties. For example, the diffusion characteristics observed inside and outside the L7 cell—i.e., faster diffusion observed inside the cell with slower diffusion in the tissue sheath surrounding the plasma membrane—shares these properties with tissues observed in MRM studies of mammalian cells^12^,^15^. By capitalizing on technological advancements in dedicated equipment for MRM including compact gradient coils^11^, which allow us to delineate cellular structures with finer temporal and spatial resolution, structural features which cannot be visualized at coarse resolutions are revealed. Such understanding is an imperative first step to developing new and ever more accurate models of tissue microstructure which in turn can be used as tools to interpret the changes brought about by pathology in the macroscopic resolution regime used for clinical MR imaging. The current study has—using state-of-the-art, commercially available RF hardware—revealed novel information regarding the MR properties of tissue microstructure at the cellular level in a well-established biological model: the isolated L7 neuron. Ultimately, it is hoped that this knowledge will aid in improving our understanding and interpretation of clinical MRI. In conjunction with high resolution protocols, cell activation through light sensitive lipochondria might open up a new chapter in functional studies on single-cells.

## Additional Information

**How to cite this article**: Lee, C. H. *et al*. Investigation of the subcellular architecture of L7 neurons of *Aplysia californica* using magnetic resonance microscopy (MRM) at 7.8 microns. *Sci. Rep*. **5**, 11147; doi: 10.1038/srep11147 (2015).

## Supplementary Material

Supplementary Information

Supplementary Information
