# Supplementary material for: Investigation of the subcellular architecture of L7 neurons of Aplysia californica using magnetic resonance microscopy (MRM) at 7.8 microns
Source: Sci Rep. 2015 Jun 10;5:11147. doi: 10.1038/srep11147 (PMC4461915; doi:10.1038/srep11147)
Supplement: Supplementary Information [file srep11147-s1.pdf]

**TITLE: Investigation of the subcellular architecture of L7 neurons of *Aplysia californica* using magnetic resonance microscopy (MRM) at 7.8 microns**

**AUTHORS**: Choong H. Lee[1,2 *], Jeremy J. Flint[1,2], Brian Hansen[3], Stephen J. Blackband[1,2,4]

## SUPPLEMENTARY INFORMATION

**Supplementary Video S1: 3D reconstruction of single *Aplysia* neuron.** Intracellular morphology and spatial continuity is evident in the 3D reconstructed data which can be viewed as nucleus in the cell (dark purple), cytoplasm (light brownish orange), and satellite cells (purple) in the extracellular matrix (green).
